# State of high-grade glioma care in Africa: insights from a scoping review of the available published literature

**DOI:** 10.3389/fonc.2026.1902240

**Published:** 2026-09-02

**Authors:** Djiby Jean Marcel Okamon, Mèhomè Wilfried Dossou, Wilfried Yao Abo, Albéric Fabrice Bocco, Thierry Alihonou, Hugues Yao Christian Dokponou, Nourou Dine Adeniran Bankole

**Affiliations:** 1 Neurosurgery Department, Yopougon Training Hospital Center, Abidjan, Côte d’Ivoire; 2 Research Department of Sub-Saharan Africa Futures Neurosurgeons Association (SAFNA), Cotonou, Benin; 3 Neurosurgery Department, Pierre Zobda Quitman Hospital, Martinique Training Hospital Center, Martinique, France; 4 Neurosurgery Department, Beaujon Hospital, AP-HP, Clichy, France; 5 Department of Neurosurgery, CNHU-HKM, Cotonou, Benin; 6 Neurosurgery Department, Caen Hospital Training Center, Caen, France; 7 Imaging Brain & Neuropsychiatry, iBrain U1253 INSERM, Université de Tours, France

**Keywords:** Africa, care, glioma, high-grade, scoping review

## Abstract

**Background:**

High-grade gliomas (HGGs), WHO grade 3–4 diffuse gliomas, are aggressive primary brain tumors. Evidence on their management and outcomes in Africa remains limited, unevenly distributed, and lacking population-based incidence data. This review mapped published literature on HGG epidemiology, clinical presentation, diagnosis, treatment, and outcomes across represented African countries.

**Methods:**

This review followed the Arksey and O’Malley framework, refined by Levac et al. and the Joanna Briggs Institute, and reported per PRISMA-ScR guidelines, with the protocol prospectively registered on OSF (osf.io/hkf6g). Six databases were searched (inception-January 2026) for studies of African patients with confirmed WHO grade 3–4 gliomas; quality was appraised by two reviewers using JBI tools. Data were synthesized descriptively and stratified by study design, with no meta-analytic pooling.

**Results:**

Twenty-five studies (2008-2025) reporting 1,212 patients from 11 of 54 African countries were included; 312 (25.7%) were excluded as non-HGG or unconfirmed, leaving 900 in the confirmed cohort. Most studies were retrospective (56%), concentrated in Morocco (36%), Nigeria, and Côte d’Ivoire (16% each); 20% were case reports or small series. Raised intracranial pressure, motor deficits, and seizures showed mean study-level frequencies of 62%, 61%, and 41% (in 14, 12, and 11 of 25 studies). MRI was reported in 80% of studies; advanced imaging was rarely available. Glioblastoma predominated (833/900, 92.6%), and molecular testing (IDH, MGMT) was inconsistent. Gross total resection was achieved in a minority of patients; no study reported intraoperative adjuncts (neuronavigation, fluorescence guidance). Mortality at last follow-up ranged from 16.1%-100% (upper value from a single four-patient case series). Median overall survival ranged from 11–14 months, closely approximating median follow-up (11.7 months); these figures should be interpreted as descriptive rather than statistically estimated survival.

**Conclusion:**

Within evidence from 11 of 54 African countries, HGG care shows major diagnostic and therapeutic disparities and regional inequalities; findings should not be generalized continent-wide. Investment in infrastructure, training, molecular diagnostics, funding, and regional collaboration is needed to improve outcomes, equity of care, and evidence representativeness.

## Introduction

High-grade gliomas (HGGs), classified as World Health Organization (WHO) grade 3 and 4 diffuse gliomas, represent the most aggressive primary brain tumors, with glioblastoma being the most prevalent and lethal subtype in adults (1, 2). These tumors are characterized by rapid cellular proliferation, diffuse invasiveness, marked angiogenesis, and a high propensity for recurrence despite multimodal treatment (3). The incidence of HGGs is reported to be lower in low- and middle-income countries (LMICs); this may reflect underdiagnosis, limited access to advanced neuroimaging, and inadequate cancer registries rather than true epidemiological variation (4). Prognosis remains poor, with median survival for glioblastoma ranging from 12 to 15 months despite aggressive therapy, while grade 3 gliomas show slightly improved outcomes depending on their molecular profile (2).

To date, no reliable population-based incidence data on HGGs exist for African countries. This reflects the near-absence of functioning, population-based cancer registries capturing central nervous system tumors across most of the continent, rather than a true absence of disease burden. This epidemiological gap is itself an important finding motivating the present review and is distinct from the descriptive evidence on the geographic distribution of published studies, histopathological subtypes, and survival that is mapped below.

Recent advancements in neuro-oncology have significantly transformed the management of HGGs in high-income settings. The integration of molecular biomarkers, including IDH mutation status, MGMT promoter methylation, 1p/19q codeletion, and EGFR amplification, has enhanced diagnostic accuracy, prognostic stratification, and therapeutic decision-making (5). The 2021 WHO CNS classification incorporates molecular alterations alongside histopathological features. Current standard-of-care treatment involves maximal safe surgical resection followed by concurrent chemoradiotherapy with temozolomide (Stupp protocol), supplemented by additional therapeutic modalities such as tumor-treating fields, immunotherapy, and targeted molecular therapies (2).

In Africa, the management of HGGs is hampered by substantial systemic and infrastructural limitations that adversely affect patient outcomes. The severe shortage of neurosurgical expertise across several African countries, often with fewer than one neurosurgeon per million inhabitants, further constrains timely surgical intervention (4). Additionally, the absence of intraoperative technologies such as neuronavigation, intraoperative MRI, fluorescence-guided surgery, and awake craniotomy for selected patients diminishes the feasibility of achieving maximal safe resection (6, 7). Pathological and molecular characterization remains limited due to the scarcity of neuropathologists and molecular diagnostic platforms; many centers continue to rely primarily on conventional histopathology without immunohistochemistry or molecular profiling. Similarly, radiotherapy infrastructure is inadequate in many regions, with limited availability of linear accelerators and advanced radiation techniques such as intensity-modulated radiotherapy and stereotactic radiotherapy (6).

Beyond infrastructural limitations, financial barriers significantly restrict access to optimal care. High out-of-pocket costs associated with surgery, chemotherapy, radiotherapy, and supportive care, combined with limited insurance coverage, frequently result in delayed, incomplete, or suboptimal treatment (8–10). The absence of structured rehabilitation programs, palliative care, and psychosocial support services further compromises quality of life and clinical outcomes (11).

Despite the growing burden of neuro-oncological diseases in Africa, data regarding the epidemiology, management strategies, and outcomes of HGGs remain scarce and fragmented, and confined to small single-center retrospective series with heterogeneous methodologies and reporting standards (4, 12, 13). A comprehensive synthesis of the available published literature is therefore necessary to identify gaps in care delivery, inform policy and resource allocation, and guide the development of context-appropriate management strategies while accurately characterizing the geographic scope of the evidence that currently exists.

Accordingly, this scoping review aims to systematically map the current published evidence on the management and outcomes of HGGs in the African countries represented in that literature, in order to support future research initiatives, strengthen healthcare systems, and advance equitable neuro-oncological care.

## Materials and methods

### Study design

This scoping review was conducted in accordance with the methodological framework proposed by Arksey and O’Malley (14), further refined by Levac et al. (15) and the Joanna Briggs Institute (JBI) (16), and reported according to the PRISMA Extension for Scoping Reviews (PRISMA-ScR) guidelines (17). The study protocol was prospectively registered on the Open Science Framework (OSF) prior to initiation (18) (registration: osf.io/hkf6g).

### Eligibility criteria

We included studies involving patients of all ages with histologically confirmed high-grade gliomas (WHO grade 3-4), encompassing glioblastoma, anaplastic astrocytoma, anaplastic oligodendroglioma, anaplastic oligoastrocytoma, and other grade 3–4 glial tumors. Eligible studies reported at least one of the following features: epidemiology, clinical presentation, diagnostic pathways, treatment (surgery, radiotherapy, systemic therapy), patient outcomes, healthcare delivery, resource utilization, or socioeconomic determinants of care.

Eligible studies were conducted in any African country, as well as multicountry studies providing disaggregated data specific to African populations. Study designs included randomized controlled trials, non-randomized studies (cohort, case-control, cross-sectional), case series, case reports, and relevant grey literature (institutional reports), in accordance with scoping review methodology. No restriction was applied regarding publication date. Only studies published in English or French were included.

We excluded studies lacking african patient data or separable african subgroups, studies focused solely on low-grade gliomas, and those not addressing epidemiology, management, or outcomes of high-grade gliomas. Editorials, commentaries without original data, conference abstracts lacking sufficient methodological detail, and studies with unextractable outcome data were also excluded.

### Information sources

A comprehensive electronic search was conducted in the following databases from inception to the date of the final search: Medline (via PubMed), Embase (via Ovid), African Journals Online (AJOL), Cochrane Library, Web of Science Core Collection, and Global Health. Additional grey literature was identified through institutional repositories and relevant organizational websites.

### Search strategy

The search strategy combined Medical Subject Headings (MeSH), controlled vocabulary, and free-text terms, structured around three main concepts: population (high-grade gliomas and related entities such as glioblastoma and anaplastic astrocytoma), geography (Africa, including individual country names as well as “Sub-Saharan Africa” and “North Africa”), and outcomes and management (treatment modalities, outcomes, and epidemiological indicators such as surgery, radiotherapy, chemotherapy, survival, mortality, incidence, and prevalence). The full electronic search strategies for each database are provided in the Appendix. The search included studies published from database inception until January 21, 2026.

### Study selection

**Figure 1:**
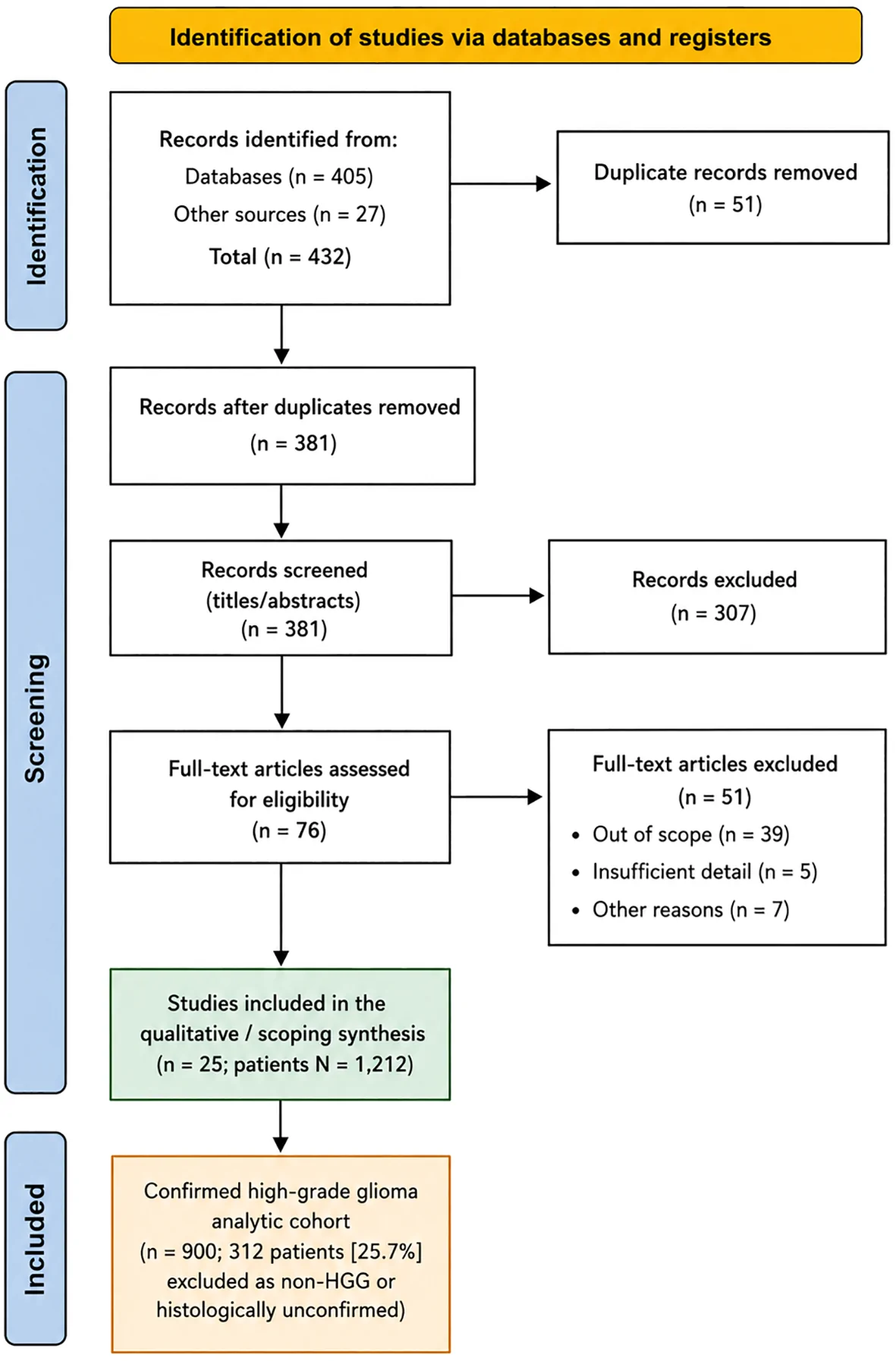
Prisma flow chart.

Records retrieved from all databases were imported into Zotero, where automated and manual deduplication was performed. The deduplicated set of records was then exported and imported into Rayyan exclusively for title and abstract screening. Five reviewers independently screened titles and abstracts using a liberal inclusion approach. Discrepancies were resolved through discussion, with arbitration by a senior reviewer (MWD or NDAB) when necessary. Full texts of potentially eligible studies were then independently assessed by two reviewers using standardized eligibility forms. Reasons for exclusion at the full-text stage were documented. The study selection process is summarized using a PRISMA 2020 flow diagram (**Figure 1**).

### Data extraction

A standardized data extraction form was developed and pilot-tested on a subset of included studies to ensure clarity and consistency. Extracted data included study characteristics (authors, year, journal, study design, study period, sample size, funding), geographic data (country and setting), patient demographics (age, sex), tumor characteristics (histology, grade, molecular markers, anatomical location), diagnostic pathways (imaging modalities, time to diagnosis, biopsy versus resection), treatment modalities (surgery, radiotherapy, chemotherapy, multimodal treatment, supportive care), outcomes (overall survival, progression-free survival, recurrence, adverse events), and health system factors (treatment delays, access to multidisciplinary care).

Data extraction occurred in two stages: a pilot phase, in which reviewers independently extracted data from five randomly selected studies to ensure consistency, and a full extraction phase, which involved refining the extraction tool based on pilot feedback. All extracted records were exported in CSV format; data management was conducted using Microsoft Excel (Microsoft Corp., Richmond, VA, USA).

### Assessment of study quality

Methodological quality was independently appraised by two reviewers using the Joanna Briggs Institute (JBI) (16) Critical Appraisal Tools corresponding to each study design (cohort/cross-sectional, case series, and case report checklists, as applicable). Disagreements in scoring were resolved through discussion between the two reviewers; a third senior reviewer (NDAB) arbitrated any disagreement that could not be resolved by consensus. This quality appraisal demonstrated an overall moderate methodological quality of the included studies. Most studies clearly described patient populations and diagnostic methods. However, common limitations included retrospective designs, small sample sizes, incomplete follow-up, and heterogeneity in outcome reporting. Quality appraisal findings were used to contextualize the interpretation of the evidence and did not influence study inclusion. Detailed appraisal results are provided in **Supplementary Material**.

### Synthesis of results

Data synthesis and data analysis are consolidated into a single subsection. Quantitative data were summarized using descriptive statistics. Categorical variables were reported as frequencies and percentages, calculated with reference to the confirmed high-grade glioma (HGG) analytic cohort (n = 900) unless otherwise specified; continuous variables were summarized using measures of central tendency and dispersion. For outcomes reported across multiple studies (presenting symptoms), we calculated the mean and range of study-level reported frequencies. These figures represent a descriptive synthesis of study-level estimates, not a patient-level pooled proportion or a formal meta-analytic estimate; consistent with scoping review methodology, no formal quantitative pooling (random-effects modelling, heterogeneity statistics) was undertaken. Outcome data were stratified by study design (cohort/cross-sectional, case series, case report) wherever feasible, and a sensitivity perspective excluding case-report-derived data is provided for mortality (see Discussion). Survival outcomes were synthesized using the medians and time-specific survival rates as reported in the primary studies; because median follow-up approximated reported median survival in several studies (see results), these figures should be interpreted as reported descriptive values rather than statistically estimated survival curves. Geographical analyses were conducted to describe variation across countries and regions. Qualitative data were analyzed using thematic synthesis to identify recurrent patterns related to healthcare delivery challenges, patient and caregiver experiences, and provider perspectives. Themes were organized within a conceptual framework describing care pathways, resource constraints, and potential leverage points for system strengthening. Extracted data were analyzed using R software for statistical computing (version 4.6.0).

## Results

### Study selection

The search identified 432 records (405 from databases, 27 from other sources). After removal of 51 duplicates via Zotero, 381 records were screened by title and abstract in Rayyan, of which 307 were excluded. The remaining 76 full-text articles were assessed for eligibility; 51 were excluded, leaving 25 studies that met the inclusion criteria (**Figure 1**).

Of the 1,212 patients identified across the 25 included studies (**Table 1**), 312 (25.7%) were excluded from the confirmed high-grade glioma analytic cohort because they represented other intracranial pathologies (meningiomas, low-grade glioma) or lacked definitive histopathological confirmation of WHO grade 3–4 glioma. The remaining 900 patients constituted the confirmed HGG analytic cohort, to which all clinical, radiological, histomolecular, and outcome analyses below refer, unless otherwise specified (**Table 2**).

**Table 1:** Geographical distribution of studies in Africa.

Countries	Studies (n)	Percentage (%)	Patients (n)	Area
Morocco	9	36	302	North Africa
Nigeria	4	16	130	West Africa
Côte d’Ivoire	4	16	71	West Africa
Algeria	1	4	333	North Africa
Tunisia	1	4	80	North Africa
Gabon	1	4	150	Central Africa
Zimbabwe	1	4	49	Southern Africa
South Africa	1	4	1	Southern Africa
Kenya	1	4	24	East Africa
Ethiopia	1	4	40	East Africa
Cameroon	1	4	32	Central Africa
Total	25	100	1 212	11 Countries

**Table 2:** Tumor profile.

Histological subtype	WHO grade	Studies numbers (n)	Cases numbers
Glioblastoma	Grade 4	18	833
Gliosarcoma	Grade 4	3	9
Anaplastic Astrocytoma	Grade 3	8	10
Anaplastic Oligodendroglioma	Grade 3	4	34
Anaplastic Ependymoma	Grade 3	2	7
Others HGG	Grade 3	1	7
Other intracranial tumors (Not-HGG)	–		312

A total of 312 patients included in the broader radiological or clinical series presented with other intracranial lesions (such as meningiomas or lesions lacking definitive histopathological confirmation of high-grade glioma). While these cases account for the total sample volume of the selected literature (n = 1,212), they were excluded from the specific HGG clinical, histomolecular, and outcome analyses to ensure the oncological validity of the review.

### Characteristics of included studies

**Figure 2:**
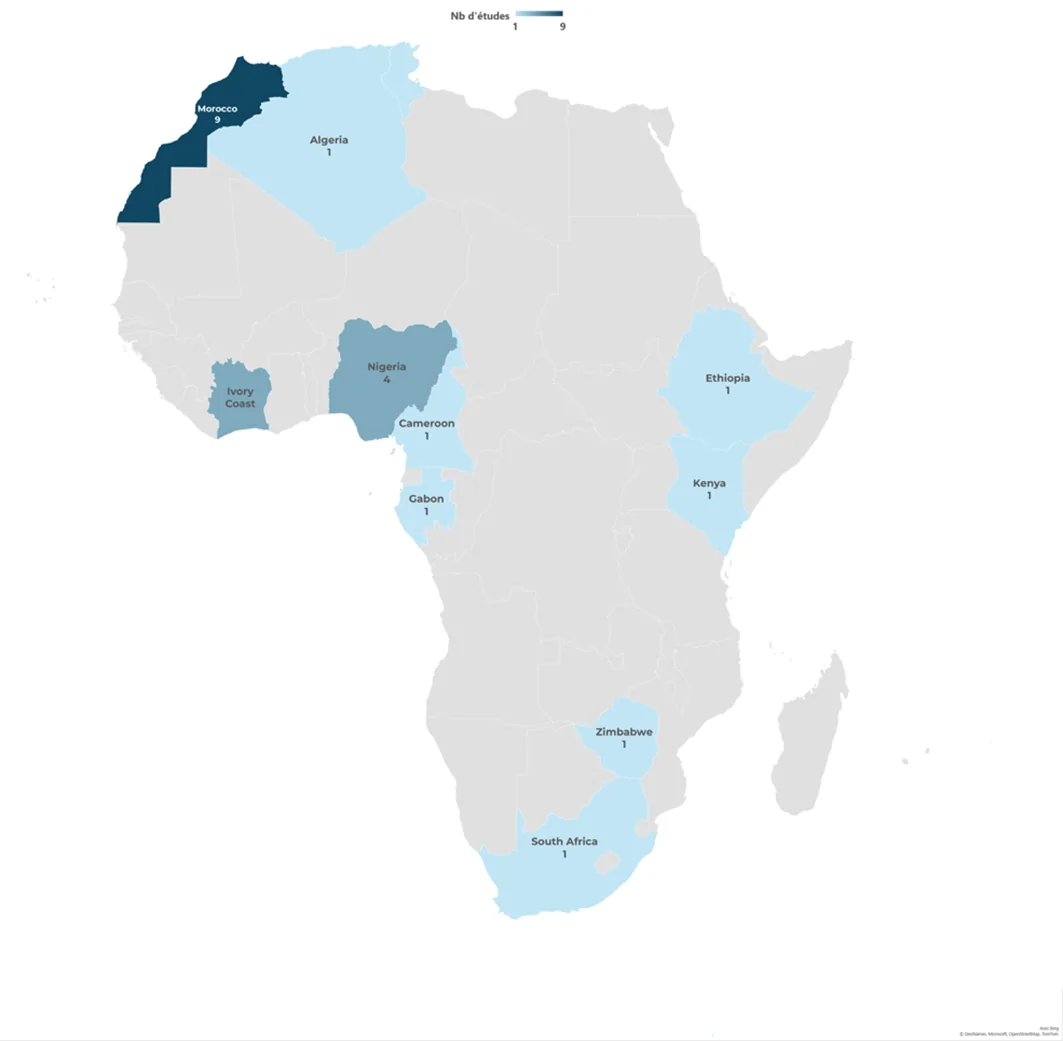
Geographic distribution of included studies across Africa.

This review included 25 studies published between 2008 and 2025 (19–43), with a peak in publication activity in 2023 (7/25 studies). These studies were conducted across 11 of the 54 african countries (**Table 1**, **Figure 2**). Morocco accounted for 9/25 studies (36.0%), while Côte d’Ivoire and Nigeria each contributed 4/25 (16.0%). Large parts of Central, East, and Southern Africa were represented by a single study each or were not represented at all; more than 40 African countries had no eligible published evidence identified by this review.

Most studies were retrospective (14/25, 56.0%), whereas 5/25 (20.0%) were case reports or small case series. Sixteen studies (64.0%) were published in indexed international journals (Medline/Scopus), and 3 (12%) in national or local journals.

### Clinical features

Raised intracranial pressure was the most frequently reported presenting symptom, observed in 14 of the 25 studies, with a mean study-level frequency of approximately 62%. Motor deficit was reported in 12 studies (mean study-level frequency = 61%), and seizures were reported in 11 studies (mean study-level frequency = 41%).

### Radiological features and imaging availability

**Figure 3:**
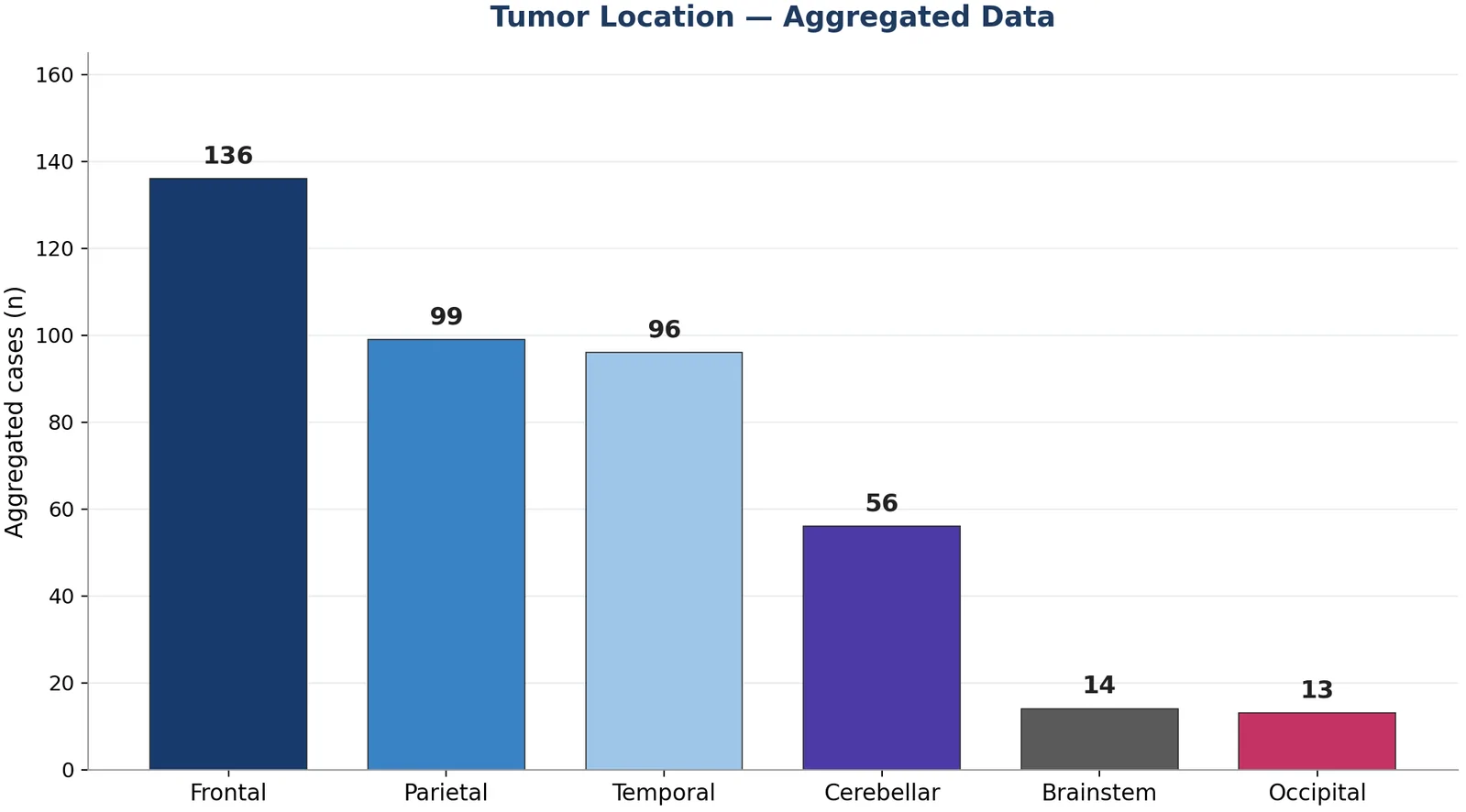
Tumor location.

MRI was reported in 20/25 studies (20–29, 31–34, 37–43), and CT scan was used in 13 studies (21–24, 26, 31, 32, 36–41, 43). Spectroscopy and tractography were reported in 3 (22, 24, 27) and 2 studies (22, 34), respectively. The frontal lobe was the most common tumor location (32.8%), followed by the cerebellum (13.5%) (**Figure 3**).

### Neurosurgical management

Gross total resection was reported in 11 studies, partial resection in 9, and biopsy in 8. Awake surgery was reported in one study, and ventriculoperitoneal shunting in two cases. Intraoperative adjuncts, including neuronavigation and 5-ALA fluorescence guidance, were not reported in any included study.

### Histopathological and molecular profiling

Histological grading data were available for all 900 confirmed HGG cases. WHO grade 4 tumors accounted for 93.6% of cases (842/900). Glioblastoma was the predominant histological subtype, comprising 833 cases (92.6% of the confirmed HGG cohort and 98.9% of WHO grade 4 tumors) (**Table 2**).

Molecular profiling according to the 2021 WHO classification was rarely reported. IDH mutation status was available in 9/25 studies, and MGMT promoter methylation status in 7/25 studies (**Table 3**). A regional gradient was observed, with North African centers more frequently reporting molecular analyses than several sub-Saharan institutions.

**Table 3:** Histomolecular profile of included studies.

Authors	Country	WHO classification	IDH	MGMT	Others molecular
Sfifou (19)	Morocco	WHO-2016	Yes	–	TP53, HIF-1α
Ga (20)	Gabon	Not specified	–	–	–
Mandour (21)	Morocco	WHO-2007	–	–	–
Ogbole (22)	Nigeria	Not specified	–	–	–
Estelle (23)	Côte d’Ivoire	Not specified	–	–	–
Touati (24)	Algeria	WHO-2007	–	–	–
Jokonya (25)	Zimbabwe	Not specified	–	–	–
Senhaji (26)	Morocco	WHO-2016	–	–	EGFR
Darfaoui (27)	Morocco	WHO-2021	Yes	Yes	–
Baba (28)	Morocco	WHO-2016	–	–	–
Oukhdouch (29)	Morocco	WHO-2021	Yes	Yes	Methylation- qPCR
Laadam (30)	Morocco	WHO-2007	–	–	–
Ndubuisi (31)	Nigeria	WHO-2016	–	–	–
Limam (32)	Tunisia	WHO-2016	–	Yes	EGFR, TP53, PTEN, MDM2
Nadvi (33)	South Africa	Not specified	–	–	–
Odukoya (34)	Nigeria	WHO-2021	Yes	Yes	TP53, ATRX, NGS, BRAF, H3K27M
Okamon (35)	Côte d’Ivoire	Not specified	–	–	–
Belghali (36)	Morocco	WHO-2016/2021	Yes	–	–
Egu (37)	Morocco	WHO-2007/2016	–	–	–
Ndubuisi (38)	Nigeria	WHO-2016	Yes	–	–
Drogba (39)	Côte d’Ivoire	Not specified	–	–	–
Drogba (40)	Côte d’Ivoire	Not specified	–	–	–
Sharma (41)	Kenya	WHO-2021	Yes	Yes	–
Gechu (42)	Ethiopia	WHO-2021	Yes	Yes	–
Nassourou (43)	Cameroon	WHO-2021	Yes	Yes	–

### Mortality and survival

Mortality reporting from 7 eligible studies was highly heterogeneous, with outcome definitions, follow-up duration, and reporting methods varying substantially. No formal pooled or weighted mortality estimate was calculated.

**Figure 4:**
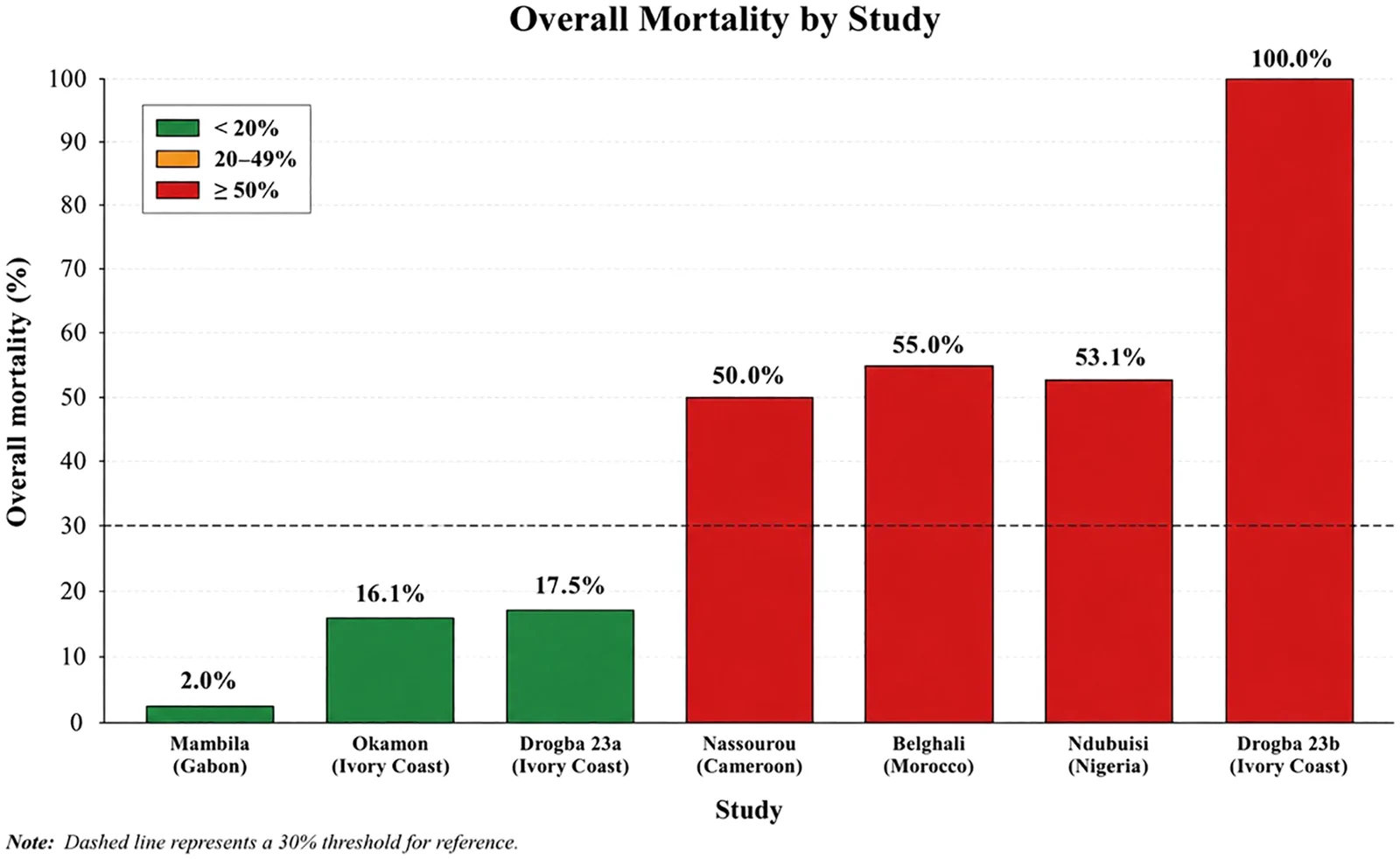
Overall mortality at last follow-up.

**Figure 5:**
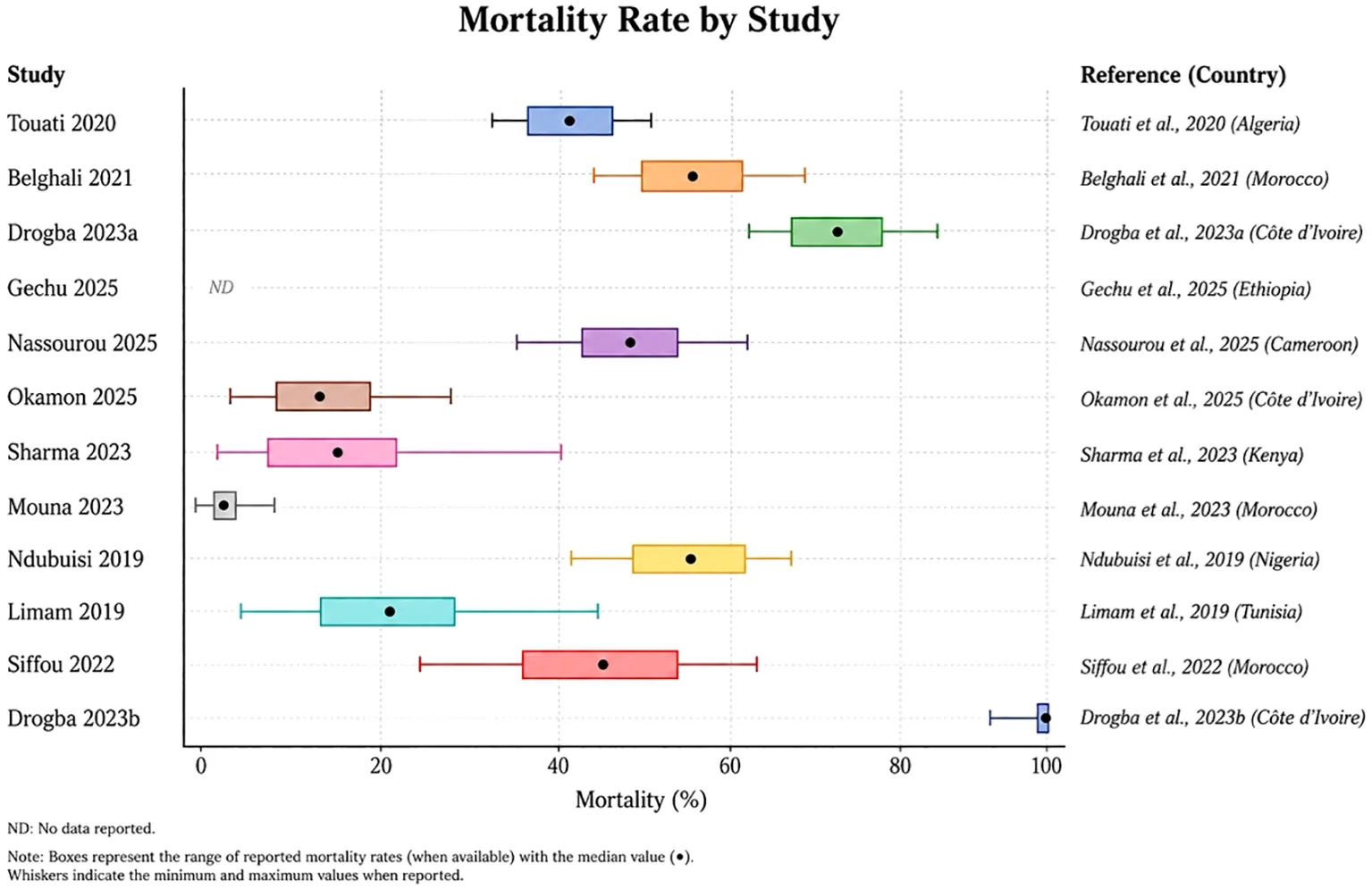
Overall survival estimation.

Reported mortality at last follow-up ranged from 16.1% to 100% (**Table 4**; **Figures 4**, **5**). The upper-bound value originates from a small case series of four patients (40) and reflects an individual cohort experience rather than a population-level estimate.

**Table 4:** Post-therapeutic follow-up of patients, recurrence and global mortality rates.

Studies	Countries	Admission average time (month)	Karnofsky score (KPS)		Median follow-up (month)	Recurrence rate (%)	Death (%)
			KPS >70%	KPS <70%			
*Touati* (24)	Algeria	–	–	–	17,4	17,4	42,9
*Belghali* (36)	Morocco	3,1 ± 3,1	–	–	13,6 ± 5,3	30,0	55,0
*Drogba* (39)	Côte d’Ivoire	–	–	–	11	10,5	73,7
*Gechu* (42)	Ethiopia	–	–	–	11,3	–	–
*Nassourou* (43)	Cameroon	2,4	–	–	10,2	–	50,0
*Okamon* (35)	Côte d’Ivoire	–	65,0%	35,0%	8 – 20	–	16,1
*Sharma* (41)	Kenya	–	–	–	6	25,0	–
*Mouna* (27)	Morocco	3,0 (1 – 5)	69%	31,0%	7	2,0	–
*Ndubuisi* (31)	Nigeria	3,1 ± 3	36,7%	63,3%	12	–	53,1
*Limam* (32)	Tunisia	–	–	–	12	24,3	–
*Sfifou* (19)	Morocco	–	72,0%	28,0%	12	46,8	–
*Drogba* (40)	Côte d’Ivoire	3,5	–	–	0,25 – 10	–	100

Reported median overall survival across studies ranged from 11 to 14 months.

Median follow-up across studies was 11.7 months (mean 11.0 months; range 5.5-17.4 months). The longest median/range follow-up was reported in Algeria (24), at 17.4 months, while the shortest follow-up periods were reported in studies from Côte d’Ivoire (1 week-10 months) (40) and Kenya (6 months) (41) (**Table 4**).

## Discussion

### Epidemiological visibility and evidence gaps

This scoping review provides one of the first syntheses of the available published evidence on HGG epidemiology, management, and outcomes in Africa. The identification of only 25 studies, reporting 900 confirmed HGG patients, from 11 of 54 countries over nearly two decades illustrates both the scarcity and the markedly uneven geographic distribution of the available literature. Evidence was concentrated in a small number of academic centers, predominantly in North and West Africa, while large parts of Central, East, and Southern Africa remain almost entirely unrepresented. This pattern most likely reflects disparities in neurosurgical capacity, neuroimaging and pathology infrastructure, research funding, and academic productivity rather than a true epidemiological difference in disease burden (4, 7, 10).

A specific and important gap concerns incidence: no population-based incidence data on HGGs were identified by this review for any African country, owing to the near-absence of functioning population-based cancer registries capturing central nervous system tumors. Consequently, the apparently lower reported burden of HGGs in the African countries studied, compared with high-income settings, should therefore be interpreted with substantial caution, since underdiagnosis, limited neuroimaging access, incomplete cancer registration, restricted neuropathology services, and mortality before specialist referral likely all contribute substantially to under-ascertainment (4, 8, 9, 44, 45).

### Clinical and diagnostic disparities

Clinically, African HGG patients present with symptom profiles consistent with global glioma biology, including raised intracranial pressure, focal neurological deficits, and seizures (2, 3, 46). The reported frequencies of these symptoms are study-level descriptive figures rather than patient-level pooled estimates, given the heterogeneity of reporting across studies. Several reports suggest delayed diagnostic trajectories characterized by prolonged symptom duration, substantial tumor burden, and impaired neurological status at presentation. These findings likely reflect multilevel barriers, including limited awareness, fragmented referral systems, specialist shortages, financial constraints, and restricted access to advanced imaging (8, 9, 45). Such delays are clinically meaningful, as preoperative functional status and tumor volume influence resectability, eligibility for adjuvant therapy, and survival (46–48).

Diagnostically, while MRI was available in many published series and CT remained widely used, advanced neuroradiological modalities central to contemporary glioma management functional MRI, perfusion imaging, tractography, and spectroscopy were rarely reported, likely reflecting equipment cost and scarcity and shortages of specialized expertise (2, 4, 6, 11, 45, 46).

Pathological and molecular characterization revealed a major gap: although glioblastoma and WHO grade 4 tumors predominated, molecular markers central to the WHO 2021 classification (IDH, MGMT, ATRX, EGFR, TERT, 1p/19q) were inconsistently reported or largely absent (1, 5, 46). This vulnerability is further illustrated by the finding that 312 of the 1,212 patients initially captured in the broader literature search (25.7%) had to be excluded from the confirmed HGG cohort, as they represented other intracranial pathologies or lacked histopathological confirmation underscoring that clinical and neuroimaging impressions alone cannot substitute for definitive tissue diagnosis in resource-constrained settings.

The included studies span three successive WHO classification eras (2007, 2016, and 2021). The absence of molecular markers in older series (24), reflects the international diagnostic standards of that time rather than a methodological shortcoming by local investigators. The persistence of purely morphological diagnosis in studies published well after 2021 highlights a widening technological gap, likely explained by limited neuropathology expertise, high testing costs, fragile laboratory infrastructure, and restricted access to sequencing platforms (4, 11, 45). A regional gradient was also observed, with North African centers reporting molecular analyses more frequently than several sub-Saharan institutions, though this should be interpreted cautiously given the limited number of studies.

### Therapeutic disparities and surgical care

Surgery remained the therapeutic cornerstone, but technologies that increasingly define modern glioma surgery neuronavigation, intraoperative MRI, 5-ALA fluorescence guidance, and awake mapping were not reported in any included study. Given the established association between extent of resection and survival in glioblastoma (49), this likely constrains surgical completeness and oncological outcomes, particularly as 5-ALA fluorescence guidance has been shown in a randomized controlled trial to significantly increase rates of complete tumor resection (50).

Radiotherapy access emerged as one of the most significant systemic bottlenecks, with severe shortages of infrastructure, unequal geographic distribution of facilities, and treatment delays constraining standard postoperative care across many countries (8, 11, 45). Chemotherapy access was similarly compromised by drug costs and procurement instability, limiting full implementation of standardized chemoradiotherapy protocols, including sustained temozolomide delivery, in many settings (9, 46, 48). These barriers mirror structural drug-access challenges documented broadly across oncology in low-resource settings (51). Supportive and rehabilitative dimensions of care neurocognitive assessment, rehabilitation, psychosocial support, and palliative care remained poorly represented in the literature, despite their relevance to a disease associated with substantial neurological morbidity.

### Outcomes and methodological considerations

Reported mortality at last follow-up ranged widely (16.1%-100%) across the studies providing outcome data; no formal pooled estimate could be reliably derived, and none is presented here. The single highest value (100%) derives from a small case series (4 patients) and should not be extrapolated to the broader confirmed HGG cohort. Excluding this case report, the upper bound narrows to 73.7% (16.1%-73.7% across the remaining six studies), though this narrower range should still be interpreted with caution given the small number of studies and the heterogeneous follow-up durations and mortality definitions across the included literature.

Reported median overall survival (11–14 months) closely approximated the median follow-up duration across studies (11.7 months), indicating that a meaningful proportion of patients were likely still alive or lost to follow-up at the time of last assessment; these figures should therefore be interpreted as reported descriptive values rather than statistically estimated survival, and likely underestimate true survival. Collectively, these findings demonstrate that the landscape of HGG care in the African countries represented in this review is shaped not only by limited epidemiological visibility but also by compounding disparities in diagnostic capability, molecular implementation, treatment access, and specialist care delivery, with retrospective designs, heterogeneous reporting, and loss to follow-up further limiting the strength of the outcome data (9, 45–48).

### Geographic scope and generalizability

It is important to emphasize that this evidence base, while the most comprehensive synthesis available to date on this topic, derives from only 11 of 54 African countries, with more than one-third of included studies originating from Morocco alone. More than 40 African countries, and large parts of Central, East, and Southern Africa specifically, are not represented in the published literature identified by this review. The findings presented here therefore characterize the available published evidence rather than HGG care across the African continent as a whole, and should not be extrapolated to unrepresented regions without caution. Publication bias likely compounds this concentration, as centers with greater clinical and research capacity, and an established publication record, are more likely to report their experience; this includes the present author group, whose related work on regional neuro-oncology networks (10) reflects a potential, non-financial academic interest disclosed in the conflict of interest statement below.

### Recommendations for future research and practice

Addressing the gaps identified in this review will require coordinated investment in neurosurgical workforce development, expansion of neuroimaging and radiotherapy capacity, and capacity building in molecular pathology. Equally important are structured research training opportunities (research methodology workshops and mentorship programs) for African neurosurgical and oncology trainees, and dedicated funding mechanisms supporting prospective, multicenter African neuro-oncology research. We specifically call for population-based and multicenter prospective studies from currently unrepresented or underrepresented regions particularly Central, East, and Southern Africa together with the establishment of multicenter registries and standardized reporting frameworks, to build a more complete and representative evidence base and to support equitable, evidence-based HGG (10, 18, 46, 51).

## Limitations

Several limitations should be considered when interpreting the findings of this scoping review. First, the evidence base derives primarily from hospital-based studies concentrated in a minority of African countries (11/54), with limited representation from Central, East, and Southern Africa; findings should not be generalized continent-wide. Second, publication bias likely affects this body of evidence, as centers with greater research capacity are more likely to publish their experience, including members of the present author group (see Conflict of Interest statement). Third, studies were conducted across three successive WHO classification eras (2007, 2016) and outcome reporting was heterogeneous, particularly regarding survival, recurrence, and mortality definitions, limiting direct comparisons between studies. Fourth, the inclusion of case reports and small case series (20% of studies) alongside cohort studies introduces potential overrepresentation of atypical presentations; the mortality and clinical-feature figures reported above should be read with this stratification in mind. Finally, consistent with scoping review methodology, this review provides a descriptive synthesis of study-level findings rather than a formal meta-analytic pooled estimate; reported survival and mortality figures should be interpreted as descriptive of the included literature rather than as precise population-level estimates.

Despite these limitations, this review provides the most comprehensive synthesis to date of high-grade glioma care in the African countries for which published evidence exists. Its methodological rigor, adherence to PRISMA-ScR and JBI recommendations, broad database coverage, and explicit stratification by study design and geographic scope offer a transparent overview of current practices, disparities, and research gaps within the represented literature.

## Conclusion

This scoping review highlights substantial challenges in high-grade glioma care across the African countries represented in the published literature, including limited epidemiological data, delayed diagnosis, inadequate access to advanced neuroimaging and molecular diagnostics, and restricted availability of standard multimodal treatments. Reported outcomes remain poor, with high perioperative mortality and wide variability in survival across studies. Marked disparities in healthcare infrastructure, particularly between North Africa and the few represented sub-Saharan countries, further contribute to inequitable care delivery. Because the available evidence covers only 11 of 54 African countries, these findings should be interpreted as reflective of the published literature rather than as representative of the continent as a whole. They nonetheless underscore the need for targeted investment, health system strengthening, expanded research training and funding, and coordinated neuro-oncology initiatives including in currently unrepresented regions to improve access to quality care and patient outcomes and the representativeness of future evidence.
