# Supplementary material for: State of high-grade glioma care in Africa: insights from a scoping review of the available published literature
Source: Front Oncol. 2026 Sep 2;16:1902240. doi: 10.3389/fonc.2026.1902240 (PMC13579621; doi:10.3389/fonc.2026.1902240)
Supplement: Supplementary file 1 [file Table1.docx]

| **No.** | **Study (First Author, Year)** | **Q1** | **Q2** | **Q3** | **Q4** | **Q5** | **Q6** | **Q7** | **Q8** | **Q9** | **Q10** | **Score** | **Quality** |
| --- | --- | --- | --- | --- | --- | --- | --- | --- | --- | --- | --- | --- | --- |
| 1 | Sfifou F. (2022) | Y | Y | Y | Y | U | Y | Y | Y | Y | Y | 9/10 | High |
| 2 | Mambila MGA (2023) | Y | Y | Y | Y | Y | U | Y | Y | Y | N | 8/10 | High |
| 3 | Mandour C. (2014) | Y | Y | NA | Y | NA | NA | Y | Y | Y | Y | 6/7 | High |
| 4 | Ogbole GI (2023) | Y | Y | Y | Y | U | Y | Y | Y | Y | N | 8/10 | High |
| 5 | Estelle A. (2023) | Y | Y | Y | Y | U | U | Y | Y | Y | N | 7/10 | Moderate |
| 6 | Touati S. (2020) | Y | Y | Y | Y | Y | Y | Y | Y | Y | Y | 10/10 | High |
| 7 | Luxwell J. (2021) | Y | Y | Y | Y | U | Y | Y | Y | Y | N | 8/10 | High |
| 8 | Senhaji N. (2017) | Y | Y | Y | Y | U | Y | Y | Y | Y | Y | 9/10 | High |
| 9 | Mouna D. (2023) | Y | Y | Y | Y | Y | Y | Y | Y | Y | N | 9/10 | High |
| 10 | Mohamed AB (2025) | Y | Y | Y | Y | Y | Y | Y | Y | Y | Y | 10/10 | High |
| 11 | Oukhdouch A. (2024) | Y | Y | Y | Y | U | Y | Y | Y | Y | Y | 9/10 | High |
| 12 | Laadam K. (2015) | Y | Y | NA | Y | NA | NA | Y | Y | Y | Y | 6/7 | High |
| 13 | Ndubuisi CA (2019) | Y | Y | Y | Y | U | Y | Y | Y | Y | N | 8/10 | High |
| 14 | Limam S. (2019) | Y | Y | Y | Y | U | Y | Y | Y | Y | Y | 9/10 | High |
| 15 | Nadvi S. (2018) | Y | Y | NA | Y | NA | NA | Y | Y | Y | Y | 6/7 | High |
| 16 | Odukoya L. (2024) | Y | Y | Y | Y | U | Y | Y | Y | Y | Y | 9/10 | High |
| 17 | Okamon DJM (2025) | Y | Y | Y | Y | Y | Y | Y | Y | Y | N | 9/10 | High |
| 18 | Belghali M. (2021) | Y | Y | Y | Y | U | U | Y | Y | Y | N | 7/10 | Moderate |
| 19 | Egu K. (2008) | Y | Y | NA | Y | NA | NA | Y | Y | Y | Y | 6/7 | High |
| 20 | Ndubuisi C. (2017) | Y | Y | Y | Y | U | Y | Y | Y | Y | N | 8/10 | High |
| 21 | Drogba L. (2023) [19 cases] | Y | Y | Y | Y | U | U | Y | Y | Y | N | 7/10 | Moderate |
| 22 | Drogba L. (2023) [4 cases] | Y | Y | Y | Y | U | NA | Y | Y | Y | Y | 7/9 | Moderate |
| 23 | Sharma K. (2023) | Y | Y | Y | Y | U | Y | Y | Y | Y | N | 8/10 | High |
| 24 | Gechu D. (2025) | Y | Y | Y | Y | Y | Y | Y | Y | Y | Y | 10/10 | High |
| 25 | Oumarou Haman N. (2025) | Y | Y | Y | Y | Y | Y | Y | Y | Y | Y | 10/10 | High |

**Table : Complete JBI Quality Assessment**

**Legend**

- Y = Yes
- N = No
- U = Unclear
- NA = Not Applicable
- Final Score = Number of “Yes” responses / Applicable questions
- Quality Level: High (≥80%), Moderate (50–79%), Low (<50%)

**JBI Criteria (Q1–Q10)**

**Q1:** Were the inclusion criteria clearly defined? (Histological/molecular diagnosis of HGG, WHO grade 3 or 4)

**Q2:** Were the study subjects and setting described in detail? (African geographical origin and tertiary-care center characteristics)

**Q3:** Was exposure measured in a valid and reliable way? (CT/MRI imaging and surgical protocols)

**Q4:** Were objective and standardized criteria used to measure the condition? (WHO Classification of Central Nervous System Tumours)

**Q5:** Were confounding factors identified? (Availability of radiotherapy, chemotherapy, and variability in healthcare resources)

**Q6:** Were strategies to deal with confounding factors stated? (Multivariable survival analyses, matching procedures)

**Q7:** Were outcomes measured in a valid and reliable way? (Karnofsky Performance Status, overall survival, recurrence)

**Q8:** Was the statistical analysis appropriate? (Kaplan–Meier analysis, Cox proportional hazards models, descriptive statistics)

**Q9:** Was the clinical and demographic description complete? (Age, sex, tumor location, extent of resection)

**Q10:** Was follow-up sufficiently reported and were losses to follow-up adequately addressed?
